# Kif4 Interacts with EB1 and Stabilizes Microtubules Downstream of Rho-mDia in Migrating Fibroblasts

**DOI:** 10.1371/journal.pone.0091568

**Published:** 2014-03-21

**Authors:** Edward J. Morris, Guilherme P. F. Nader, Nagendran Ramalingam, Francesca Bartolini, Gregg G. Gundersen

**Affiliations:** Department of Pathology and Cell Biology, Columbia University, New York, New York, United States of America; University of Illinois at Chicago, United States of America

## Abstract

Selectively stabilized microtubules (MTs) form in the lamella of fibroblasts and contribute to cell migration. A Rho-mDia-EB1 pathway regulates the formation of stable MTs, yet how selective stabilization of MTs is achieved is unknown. Kinesin activity has been implicated in selective MT stabilization and a number of kinesins regulate MT dynamics both *in vitro* and in cells. Here, we show that the mammalian homolog of *Xenopus* XKLP1, Kif4, is both necessary and sufficient for the induction of selective MT stabilization in fibroblasts. Kif4 localized to the ends of stable MTs and participated in the Rho-mDia-EB1 MT stabilization pathway since Kif4 depletion blocked mDia- and EB1-induced selective MT stabilization and EB1 was necessary for Kif4 induction of stable MTs. Kif4 and EB1 interacted in cell extracts, and binding studies revealed that the tail domain of Kif4 interacted directly with the N-terminal domain of EB1. Consistent with its role in regulating formation of stable MTs in interphase cells, Kif4 knockdown inhibited migration of cells into wounded monolayers. These data identify Kif4 as a novel factor in the Rho-mDia-EB1 MT stabilization pathway and cell migration.

## Introduction

Rearrangements of microtubules (MTs) play a central role in the establishment of cell polarity in many systems [1]. In migrating cells, MTs contribute to the front-back polarity that is essential for directional migration of cells in a variety of environments. MTs are thought to provide the tracks for directional delivery of membrane precursors and actin regulators necessary for protrusion of the leading edge [2], [3], [4]. MTs also regulate the turnover of focal adhesions by stimulating the disassembly of focal adhesions through endocytic processes [5], [6], [7], [8]. In addition, MTs regulate myosin contraction in the cell rear in certain migrating cells such as neutrophils and T cells [9], [10].

To contribute to front-back polarity in migrating cells, the MT array itself becomes polarized. Several sources of MT polarization in migrating cells have been identified. Radial MT arrays are biased toward the front of many migrating cells by the specific orientation of the centrosome toward the leading edge [11]. The oriented centrosome positions the associated Golgi and endocytic recycling compartment to direct vesicular traffic toward the leading edge. The reorientation of the Golgi may also reinforce MT asymmetry toward the leading edge as the Golgi itself can nucleate MTs in certain cell types [3]. Factors that interfere with centrosome orientation usually reduce the rate of cell migration [12], [13], [14], although direct laser ablation of the centrosome has modest-to-strong effects on cell migration depending on the cell type [15], [16].

A second source of MT polarization is the selective stabilization of a subset of MTs oriented toward the cell's leading edge [1], [17]. Because of their longevity, these selectively stabilized MTs become post-translationally modified by detyrosination and/or acetylation of tubulin. Even in situations where the centrosome does not orient toward the leading edge, for example, in a subset of fibroblasts migrating in 2D or in fibroblasts migrating on fibrillar 1D matrices, MT stabilization remains highly biased toward the front of the cell [17], [18], [19], [20]. Post-translationally modified MTs are longer-lived than their dynamic counterparts [21], [22] and serve as preferred tracks for certain kinesin motors [23], [24], [25], [26], [27], [28]. Thus, the generation of selectively stabilized MTs biases vesicle trafficking toward the leading edge in migrating cells.

Posttranslational modification of MTs may contribute to their stability [29], yet studies have shown that this is not likely responsible for the initial generation of stability of the long-lived MTs. Posttranslational modification of tubulin within MTs is relatively slow compared to dynamic turnover of MTs and in starved NIH3T3 fibroblasts stimulated with the serum factor lysophosphatidic acid (LPA), MTs are stabilized within minutes, long before the accumulation of posttranslational detyrosination [30]. In addition, treatments that enhance the levels of detyrosinated or acetylated tubulin do not directly lead to stabilized MTs [31], [32], [33].

Factors have been identified that contribute to the selective stabilization of MTs in cells. Rho GTPase and its downstream effector the formin mDia are key factors in a MT stabilization pathway that mediates the selective stabilization of MTs in migrating fibroblasts [31], [34], [35] and other cell types [36], [37], [38], [39]. Rho only stimulates mDia in the presence of integrin and FAK signaling, which may restrict the formation of stable MTs to the leading edge [40]. mDia interacts with three MT +TIP proteins, EB1, APC and CLIP170 and the interactions with EB1 and APC have been implicated in MT stability [38], [41], [42]. In vitro, mDia2 binds directly to MTs and can stabilize them against cold-induced depolymerization, although it does not generate nondynamic MT ends typical of selectively stabilized MTs in vivo (see below) [43]. mDia and other formins have recently emerged as MT regulators in addition to their role in regulating actin nucleation and elongation [44], [45]. Other factors, including two other +TIPs CLASP and ACF7/MACF [37], [46], actin capping protein [47], and the negative regulator moesin [48] and are also involved in the generation of selectively stabilized MTs. In addition to the Rho-mDia-EB1 MT stabilization pathway, other MT stabilization pathways have been described [49], [50].

An unusual property of selectively stabilized MTs that may explain their longevity is the inability of their plus ends to add or lose tubulin subunits [22], [34], [40], [51]. Indeed, these MTs behave as if their ends are capped, a property that may also explain their resistance to MT antagonists and to dilution after detergent permeabilization of cells [32], [51]. The nature of this putative cap is unknown. Some of the factors functioning in the MT stabilization pathway have been localized to the ends of stable detyrosinated MTs [42], yet none of these factors have been shown to directly cap MTs to convert them to nondynamic MTs. A study with permeabilized cell models showed that the putative capping activity of stabilized MTs has characteristics of kinesin motor proteins, including inhibition by the non-hydrolyzable ATP analog AMP-PNP [51]. Here we tested the possibility that kinesin motor proteins may be involved in the generation of selective MT stability in cells. Among a group of kinesins implicated in MT stability, we identify Kif4 as a novel factor in the selective stabilization of MTs in migrating cells and provide evidence that this protein functions downstream of other proteins in the Rho-mDia MT stabilization pathway and contributes to cell migration.

## Results

### Kif4 motor domain induces stable MT formation *in vivo*

We first tested whether kinesins can induce the formation of selectively stabilized MTs by expressing motor domains of kinesins in serum-starved NIH3T3 fibroblasts that have low levels of stable MTs as judged by the lack of detyrosinated and nocodazole resistant MTs [30], [34], [42], [52]. Throughout this paper we refer to stable MTs with high levels of detyrosinated tubulin as Glu MTs (reflecting the newly exposed glutamate residue formed by removal of tyrosine from the C-terminus of α-tubulin) and their dynamic counterparts as Tyr MTs. We tested kinesins that have been implicated in MT stability based upon: 1) their interaction with known microtubule stabilizing factors (Kif3, a kinesin 2 which binds APC) [53], 2) their ability to stabilize MTs in epithelial cells (Kif17, another kinesin 2) [54]; or 3) their ability to render MTs nondynamic in vitro (Kif4, a kinesin 4 and ortholog of Xenopus XKLP1) [55], [56] and in spindle midzone MTs [57]. We were particularly interested in testing Kif4, because the motor domain of XKLP1 prevents tubulin subunit addition to or lose from MTs in biochemical studies [56]. We chose not to explore a possible role for kinesin-8 motors (such as Kif18A), which also regulate MT dynamics, as they seem to primarily affect spindle MTs and do not appear to stabilize MTs against antagonists [58], [59], [60].

**Figure 1:**
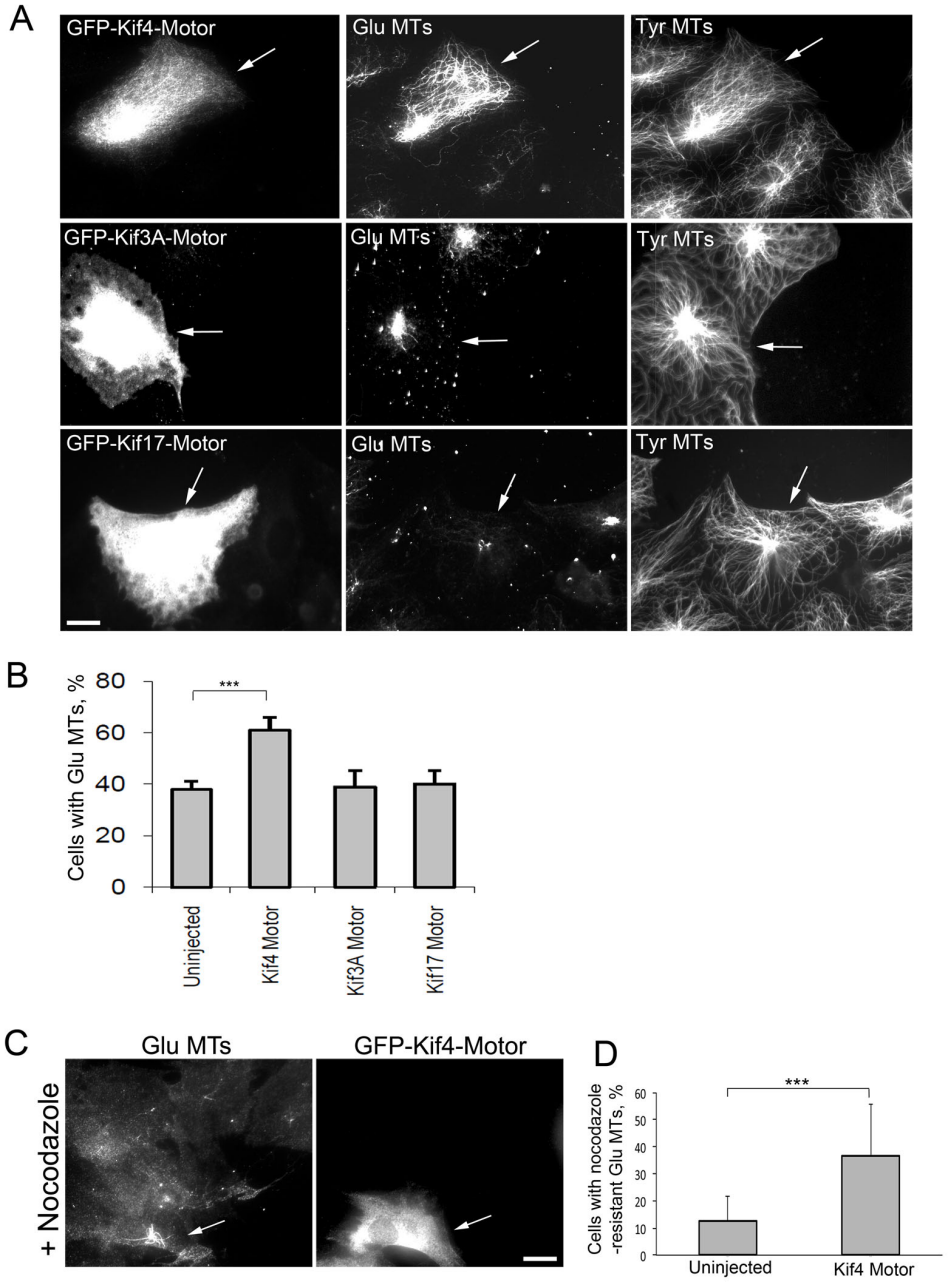
Kif4 motor domain induces the formation of stable Glu MTs in starved NIH3T3 fibroblasts. A) Immunofluorescence of Glu MTs and Tyr MTs in starved NIH3T3 fibroblasts expressing the indicated GFP-tagged kinesin motor constructs. Arrows indicate expressing cells. B) Quantification of Glu MT formation in starved NIH3T3 fibroblasts expressing the indicated kinesin motors. n>70 cells; error bars, SEM from at least 6 experiments. C) Immunofluorescence staining of Glu MTs in GFP-Kif4 motor expressing NIH3T3 fibroblasts treated with 10 µM nocodazole for 1 hr. The expressing cell (arrow) has nocodazole-resistant Glu MTs. D) Quantification of cells with nocodazole resistant Glu MTs. Error bars, SEM from 3 experiments. Bars: A, C 10 μm.

Green fluorescent protein (GFP)-tagged constructs encoding the motor domain of these kinesins were microinjected into nuclei of starved NIH3T3 fibroblasts bordering an in vitro wound and after 2 hr of expression, levels of Glu MTs were assessed in fixed cells by immunofluorescence. The motor domain of Kif4 induced Glu MTs in serum-starved NIH3T3 fibroblasts compared to uninjected neighboring cells (Figure 1A, B). The Kif4 motor domain induced only a subset of the MTs to become Glu MTs and did not detectably alter the distribution of Tyr MTs, consistent with it selectively, rather than globally stabilizing MTs. Glu MTs in the Kif4 expressing cells were preferentially oriented toward the leading edge (as in Figure 1A) in 70 +/− 7% (N = 3) of the cells, similar to the response of starved NIH3T3 fibroblasts to serum, LPA or active Rho [30], [52]. Kif3 or Kif17 motor domains did not induced the formation of Glu MTs above background levels when expressed in starved cells under identical conditions, even though the proteins were expressed at comparable levels to Kif4 as judged by GFP fluorescence (Figure 1A, B).

Glu MT staining is widely used as a marker for MT stability, but it was formally possible that Kif4 altered the enzymatic removal of tyrosine from α-tubulin instead of directly stabilizing MTs. To test this possibility and as an independent test of MT stabilization, cells expressing GFP-Kif4 motor domain were treated with nocodazole to depolymerize dynamic MTs and then stained for Glu tubulin. Starved NIH3T3 fibroblasts expressing GFP-Kif4 motor domain had numerous nocodazole-resistant Glu MTs whereas uninjected cells had only one or two short nocodazole-resistant MTs (Figure 1C, D). We conclude that the motor domain of Kif4, but not that of several other kinesins, is sufficient to induce the formation of stabilized and posttranslationally modified MTs in starved NIH3T3 fibroblasts.

### Kif4 is required for LPA-induced formation of Glu MTs

**Figure 2:**
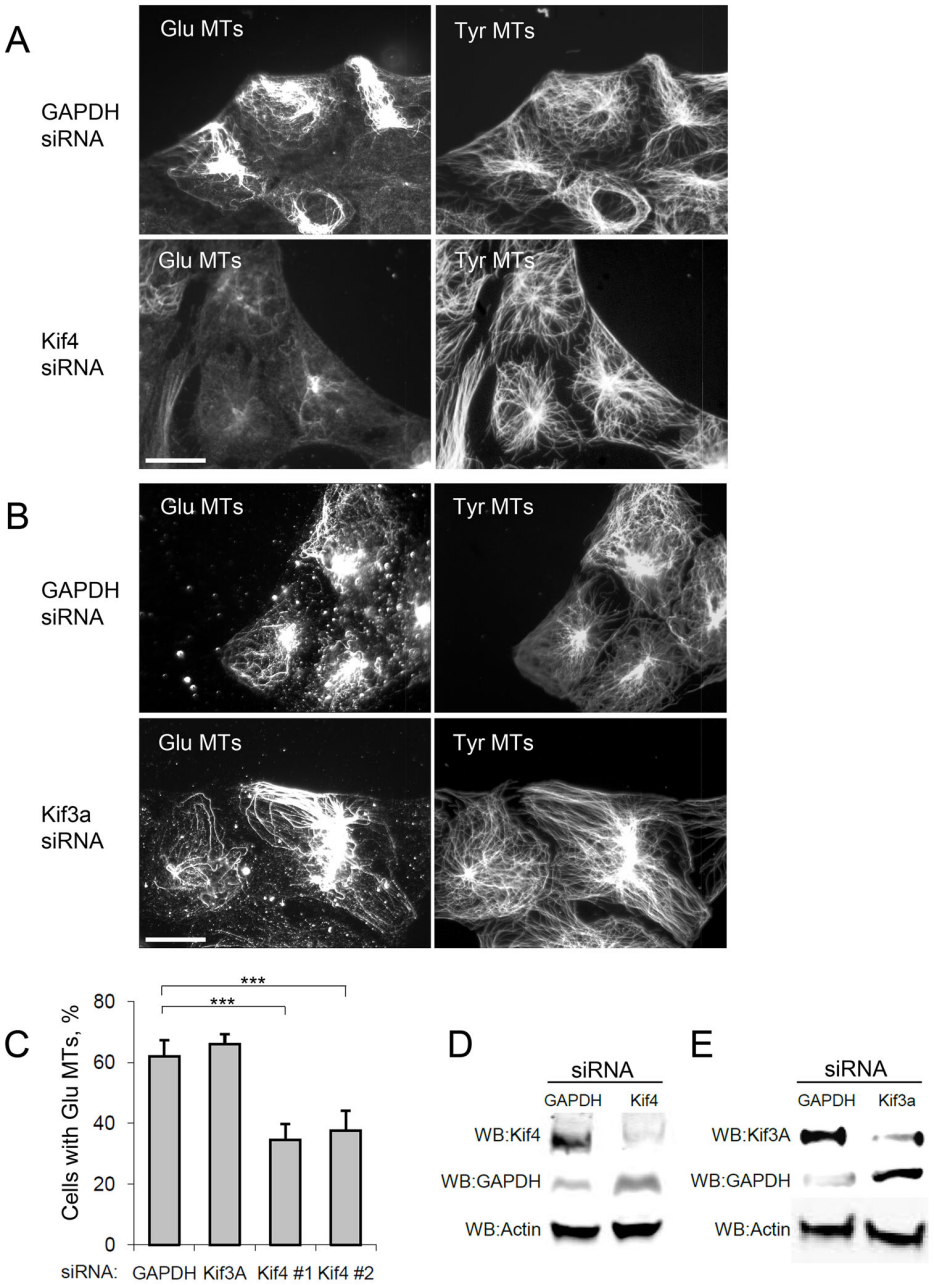
Knockdown of Kif4 inhibits LPA-induced formation of Glu MTs in NIH3T3 fibroblasts. A, B) Glu MT and Tyr MT staining of LPA-stimulated NIH3T3 fibroblasts transfected with the indicated siRNAs. C) Quantification of the percent of siRNA-treated cells that scored positive for Glu MTs. Two different siRNAs targeting Kif4 (#1 and #2) gave similar results. n>100 cells; error bars, SEM from at least 5 experiments. D, E) Western blots of NIH3T3 fibroblasts treated with indicated siRNAs and blotted for the indicated proteins. Quantification of the bands revealed over 70% knockdown of the indicated kinesins. Bars: A, B, 20 μm.

To test whether Kif4 was necessary for formation of Glu MTs, we depleted Kif4 with small interfering RNAs (siRNAs) and then induced Glu MTs in serum-starved NIH3T3 fibroblasts by treating with the serum factor LPA. As controls, we depleted either glyceraldehyde 3-phosphate dehydrogenase (GAPDH) or Kif3A (we note that we were unable to test the role of Kif17, as it is not expressed in NIH3T3 fibroblasts, see Figure S1 in File S1). Kif4 depletion inhibited LPA-induced Glu MT formation while control siRNAs had no effect (Fig. 2A–C). Kif4 depletion had no noticeable effects on Tyr MTs (Figure 2A), suggesting that it did not affect dynamic MTs. Knockdown of kinesins was verified by western blot, which showed that Kif4 and Kif3A were knocked down approximately 70% compared to GAPDH (control) siRNA-treated cells (Figure 2D, E). A second siRNA sequence to Kif4 also blocked Glu MT formation limiting the possibility that the effects of the Kif4 siRNAs were due to off-target effects (Figure 2C and Figure S2 in File S1). While Kif4 depletion inhibited Glu MT formation, it did not affect LPA-induced actin stress fiber formation (Figure S3 in File S1). These results show that Kif4 is necessary for LPA-induced formation of Glu MTs and suggest that it specifically regulates MTs rather than actin filaments downstream of LPA stimulation.

### Endogenous Kif4 localizes to the ends of Glu MTs

**Figure 3:**
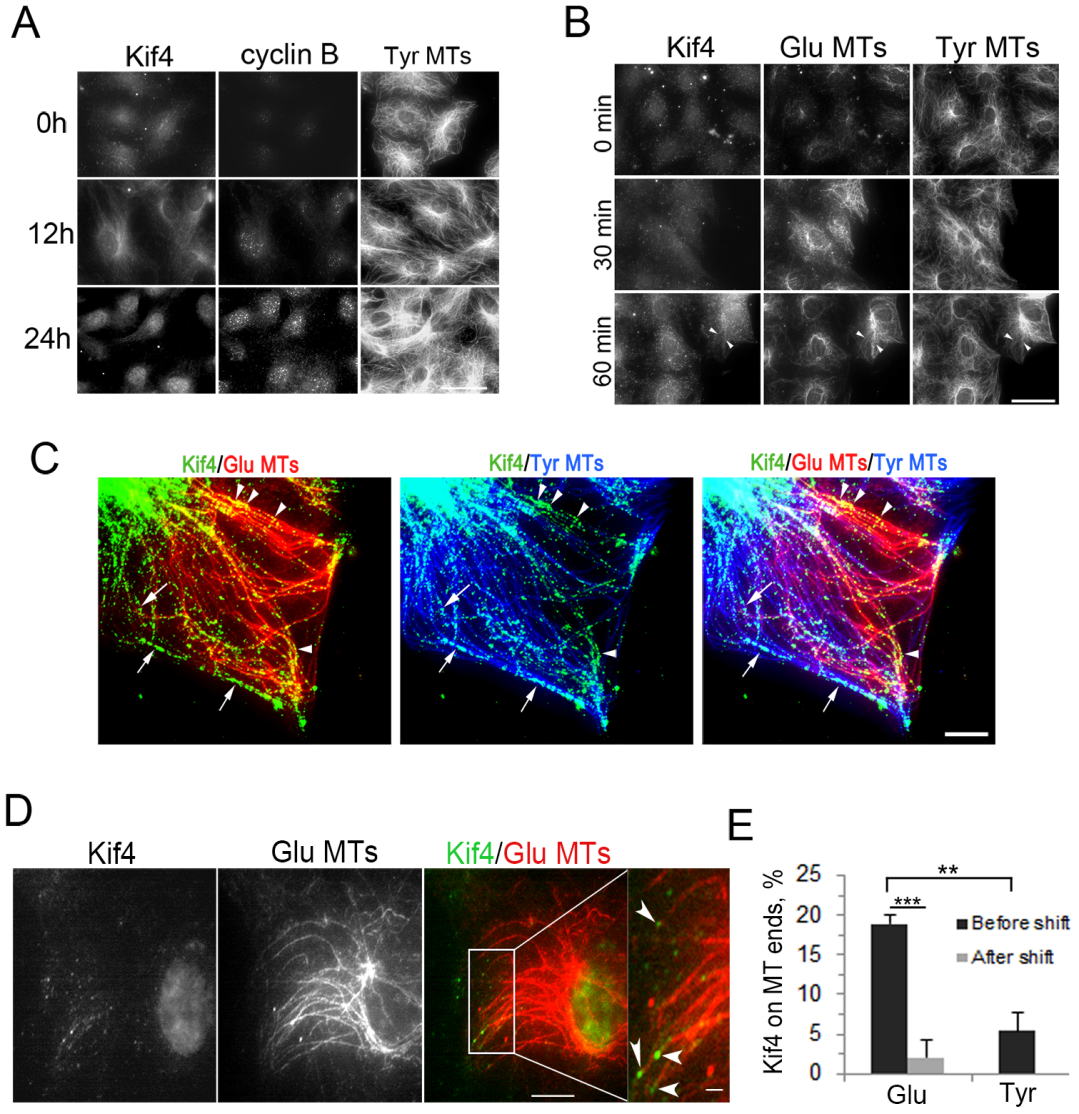
Localization of endogenous Kif4 in interphase cells. A) Immunofluorescence images of Kif4, cyclin B and Tyr MTs in serum-starved NIH3T3 fibroblasts (0 h) and in cells stimulated with serum for 12 and 24 h. B) Immunofluorescence images of Kif4, Glu and Tyr MTs in serum-starved NIH3T3 fibroblasts (0 min) and in cells stimulated with LPA for 30 and 60 min. Arrowheads indicate linear accumulations of Kif4 that coaligned with Glu and Tyr MTs. C) TIRF immunofluorescence images of Kif4, Glu and Tyr MTs in serum-stimulated NIH3T3 fibroblasts. Linear accumulations of Kif4 on Glu MTs are indicted by arrowheads; on Tyr MTs by arrows. D). TIRF immunofluorescence images of Kif4 localization on Glu MT ends. The boxed region in the merged image is shown at higher magnification in the right panels. E) Quantification of Kif4 on Glu and Tyr MT ends in serum-stimulated NIH3T3 fibroblasts. To account for random colocalization, overlaid Kif4 images were shifted relative to Glu MT images and then recounted. n>50 ends, error bars, SEM from three experiments. Bars: A, B, 20 μm; C, 5 μm. D, 10 µm; 5 µm (high mag).

We localized endogenous Kif4 to determine if it associated with Glu MTs. Kif4 has been described as a chromokinesin and much of Kif4 is localized in the nucleus before mitosis [61], [62]. Because of this, we first checked if Kif4 was present in the cytoplasm of serum-stimulated starved NIH3T3 fibroblast and whether its nuclear localization was regulated during the cell cycle. In starved cells or at early times after serum stimulation, there was little detectable Kif4 in the nucleus and diffuse staining of Kif4 in the cytoplasm; at 12–24 hr of serum stimulation, corresponding to late G1/S and G2 phases, Kif4 appeared in both the cytoplasm and the nucleus suggesting that Kif4′s nuclear localization is cell cycle regulated (Figure 3A). The cytoplasmic staining of Kif4 in unstimulated cells, which mostly appeared punctate, was dramatically reduced by siRNA–mediated depletion of Kif4 (Figure S4 in File S1), indicating that the signal detected with the Kif4 antibody was specific. In LPA-treated cells, Kif4 cytoplasmic staining appeared to increase coincident with the formation of Glu MTs and in some cells appeared as linear accumulations that paralleled MTs (Figure 3B).

To address Kif4′s localization further and in particular to probe whether Kif4 might be associated with Glu MTs, we used total internal reflection fluorescence (TIRF) microscopy. Most of the Kif4 puncta observed by TIRF microscopy were associated with MTs with linear accumulations detected on both Glu and Tyr MTs (Figure 3C). We were particularly interested in the ends of Glu MTs, because localization at this site is readily quantifiable and because other factors in the Rho-mDia pathway are localized on the ends of Glu [38], [42]. In serum-stimulated NIH3T3 fibroblasts, Kif4 puncta were detected on a number of Glu MT ends and also along their length (Figure 3D, E). In contrast, fewer Kif4 puncta were localized on Tyr MT ends (Figure 3D, E). To account for random localization, we determined the number of Kif4 puncta on Glu and Tyr MTs ends before and after shifting the Kif4 image relative to the MT images: for both types of MTs, shifting the images eliminated the colocalization with the ends, indicating that the Kif4 localization on MT ends was not due to random overlap of Kif4 puncta with MT ends. A similar analysis of Kif4 localization in TC-7 cells, which have particularly distinct Glu MTs, also revealed specific localization of Kif4 on Glu MT ends (Figure S5 in File S1). These results show that endogenous Kif4 specifically accumulates on some Glu MTs ends, consistent with a direct involvement of Kif4 in MT stabilization.

### Kif4 is required for induction of Glu MTs by factors in the Rho-mDia-EB1 MT stabilization pathway

**Figure 4:**
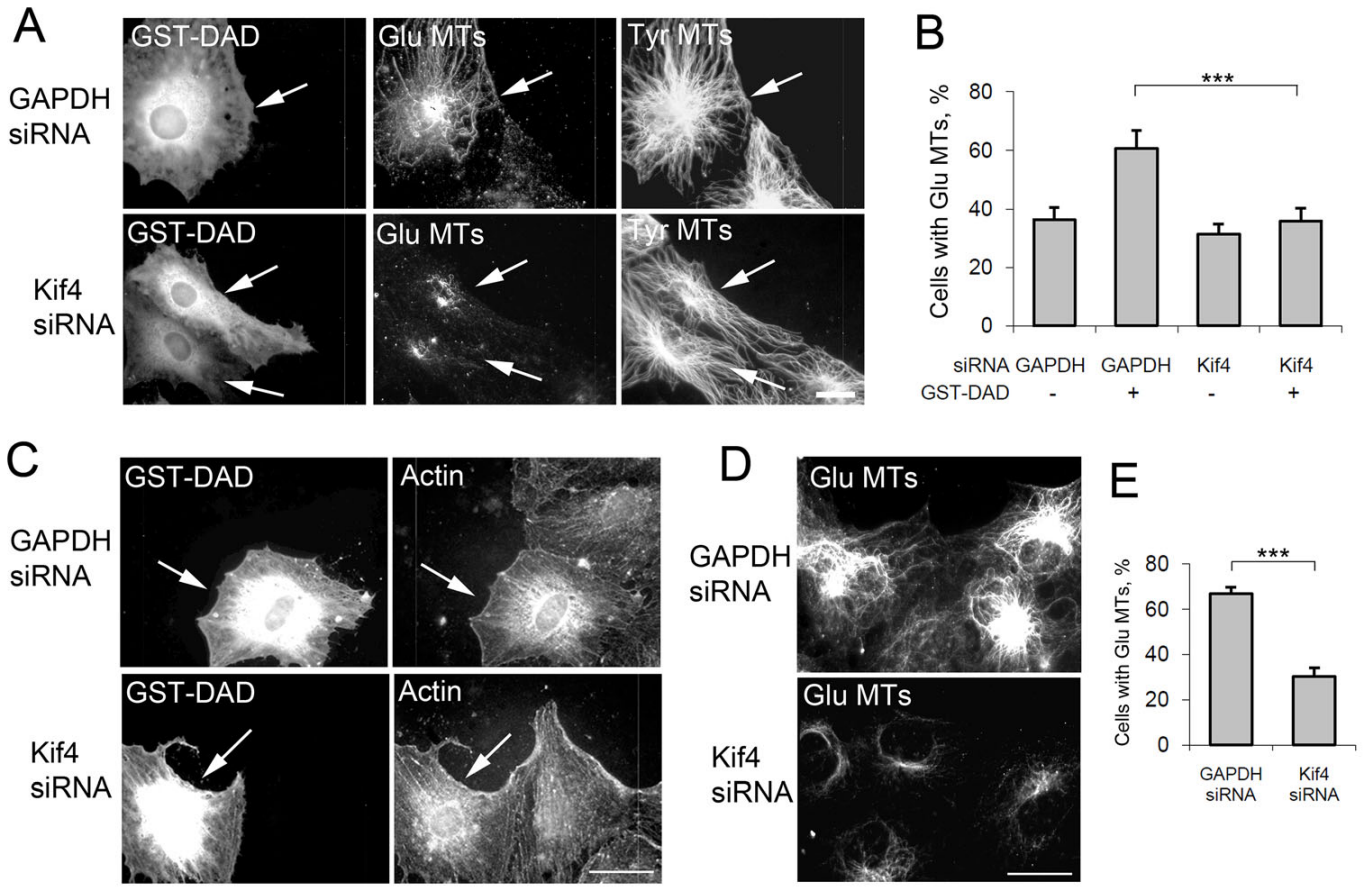
Kif4 functions downstream of mDia in the Rho-mDia-EB1 MT stabilization pathway. A) Immunofluorescence staining of Tyr MTs and Glu MTs in NIH3T3 fibroblasts treated with GAPDH (control) siRNA or Kif4 siRNA and microinjected with GST-DAD. Arrows indicate injected cells. B) Quantification of GST-DAD induction of Glu MTs in siRNA treated cells. n>50 cells; error bars, SEM from at least 4 experiments. C) Immunofluorescence staining of GST-DAD and phalloidin staining of F-actin in NIH3T3 fibroblasts treated with GAPDH (control) or Kif4 siRNA and microinjected with GST-DAD (arrows). D) Immunofluorescence staining of Kif4 and Glu MTs in starved NIH3T3 fibroblasts treated with indicated siRNAs and 10 mM LiCl for 2 hr. E) Quantification of LiCl-induced Glu MTs in GAPDH control and Kif4 siRNA cells. n>50 cells; error bars, SEM from at least 4 experiments. Bars: A, D 10 μm; C 20 μm.

To test the relationship between Kif4 and the Rho-mDia-EB1 MT stabilization pathway, we asked if Kif4 was necessary for the induction of Glu MTs stimulated by known intracellular activators of the pathway. The formation of Glu MTs in serum starved NIH3T3 fibroblasts can be stimulated by expressing the Dia autoregulatory domain (DAD) of mDia, which relieves the autoinhibition of the formin and activates it toward both actin and MTs [34], [40]. Microinjection of GST-DAD into serum-starved NIH3T3 fibroblasts depleted of Kif4 did not induce Glu MT formation, whereas it did when introduced into control (GAPDH) depleted cells (Figure 4A, B). While GST-DAD failed to induce Glu MTs in Kif4 depleted cells, it still stimulated actin cable formation showing that Kif4 depletion did not prevent DAD from activating mDia (Figure 4C).

To test further whether Kif4 functioned downstream of mDia in the formation of Glu MTs, we tested whether Kif4 was necessary for the induction of Glu MTs in serum starved NIH3T3 fibroblasts treated with LiCl, an inhibitor of GSK-3β. Activation of mDia by Rho leads to the inhibition of GSK-3β and this is necessary for the formation of Glu MTs in NIH3T3 fibroblasts [63]. LiCl treatment of NIH3T3 fibroblasts depleted of Kif4 failed to induce the formation of Glu MTs, whereas similar treatment of control (GAPDH) depleted cells did (Figure 4D, E). Combined, these results suggest that Kif4 functions downstream of mDia in Glu MT formation and that Kif4 is not involved in mDia's stimulatory effect on actin filaments. Consistent with this interpretation, we did not detect a significant alteration in the distribution of mDia1 or EB1 in GFP-Kif4 motor expressing cells (Figure S6 in File S1).

### Kif4 and EB1 require each other to generate stable MTs

**Figure 5:**
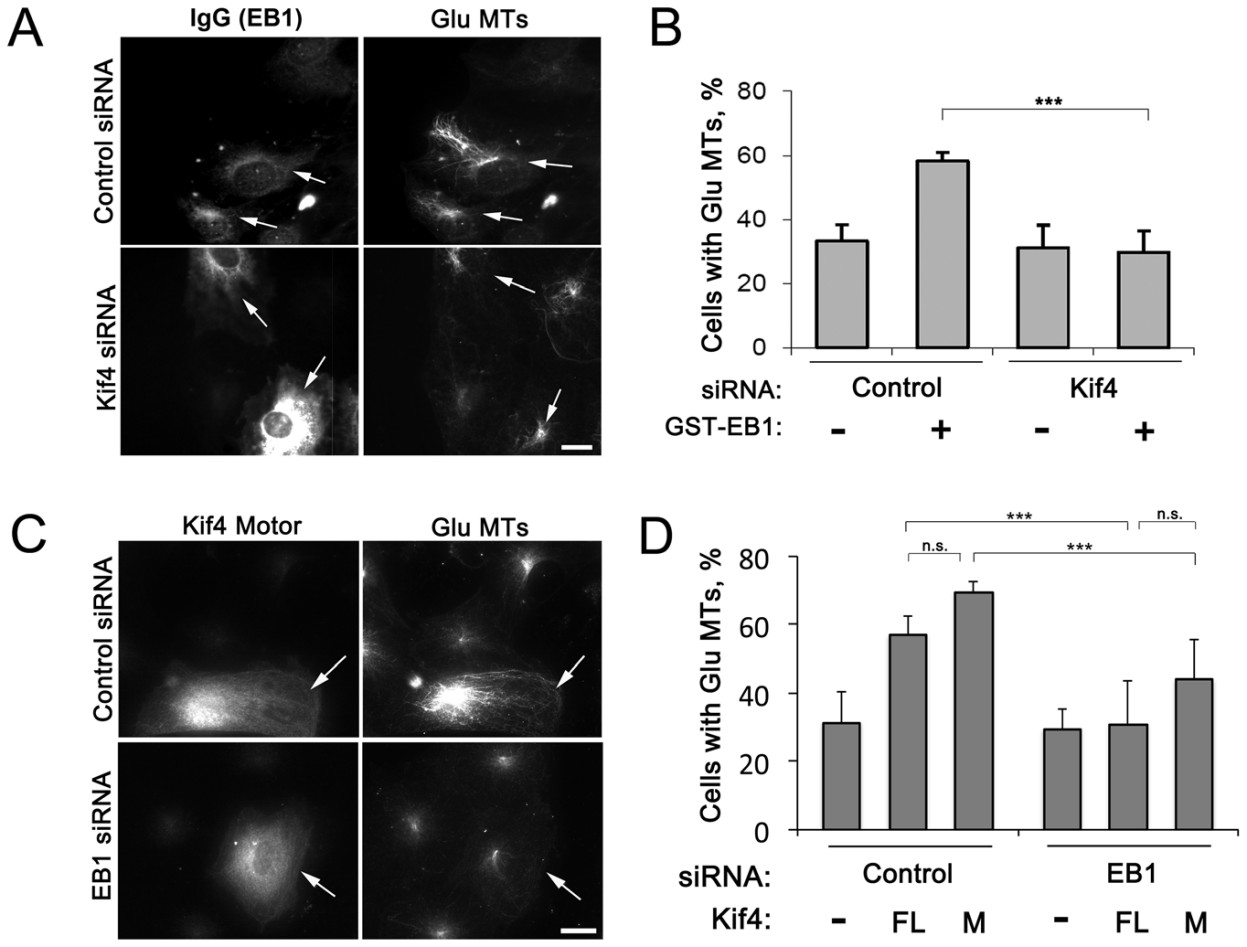
Expressed Kif4 and EB1 require each other to induce Glu MTs. A) Immunofluorescence staining of Glu MTs in starved NIH3T3 fibroblasts treated with control (GAPDH) siRNA or Kif4 siRNA and microinjected with GST-EB1 (arrows). Human IgG (IgG) was used as an injection marker for GST-EB1 injected cells. Arrows indicate injected cells. B) Quantification of the percentage of siRNA-treated cells exhibiting Glu MTs after injection with GST-EB1 protein. n>100 cells; error bars are SEM from 4 experiments. C) Immunofluorescence staining of Glu MTs in starved NIH3T3 fibroblasts treated with control (noncoding) siRNA or EB1 siRNA and expressing GFP-Kif4 motor (arrows). D) Quantification of the percentage of siRNA-treated cells exhibiting Glu MTs after expression of GFP-Kif4 full length (FL) or motor (M) constructs. n>100 cells; error bars, SEM from at least 4 experiments. Bars: A, C, 10 μm.

EB1 functions downstream of mDia in the MT stabilization pathway and overexpression of EB1 induces the formation of stable MTs in serum-starved NIH3T3 fibroblasts [42]. We tested if the induction of Glu MTs by Kif4 and/or EB1 expression in starved NIH3T3 fibroblasts depended on each other. Kif4 or control (GAPDH) depleted serum-starved NIH3T3 fibroblasts were microinjected with GST-EB1 and the formation of Glu MTs was assessed. GST-EB1 induced Glu MTs in control cells, but not in Kif4 depleted cells (Figure 5A, B). Similarly, expression of either GFP-tagged Kif4 motor domain or Kif4 full length in starved cells knocked down for EB1 (Figure S7 in File S1), which inhibits Glu MTs induced by LPA [42], did not induce Glu MTs (Figure 5C, D). These results suggest that Kif4 and EB1 are mutually dependent for inducing stable MTs.

### Kif4 interacts directly with EB1

**Figure 6:**
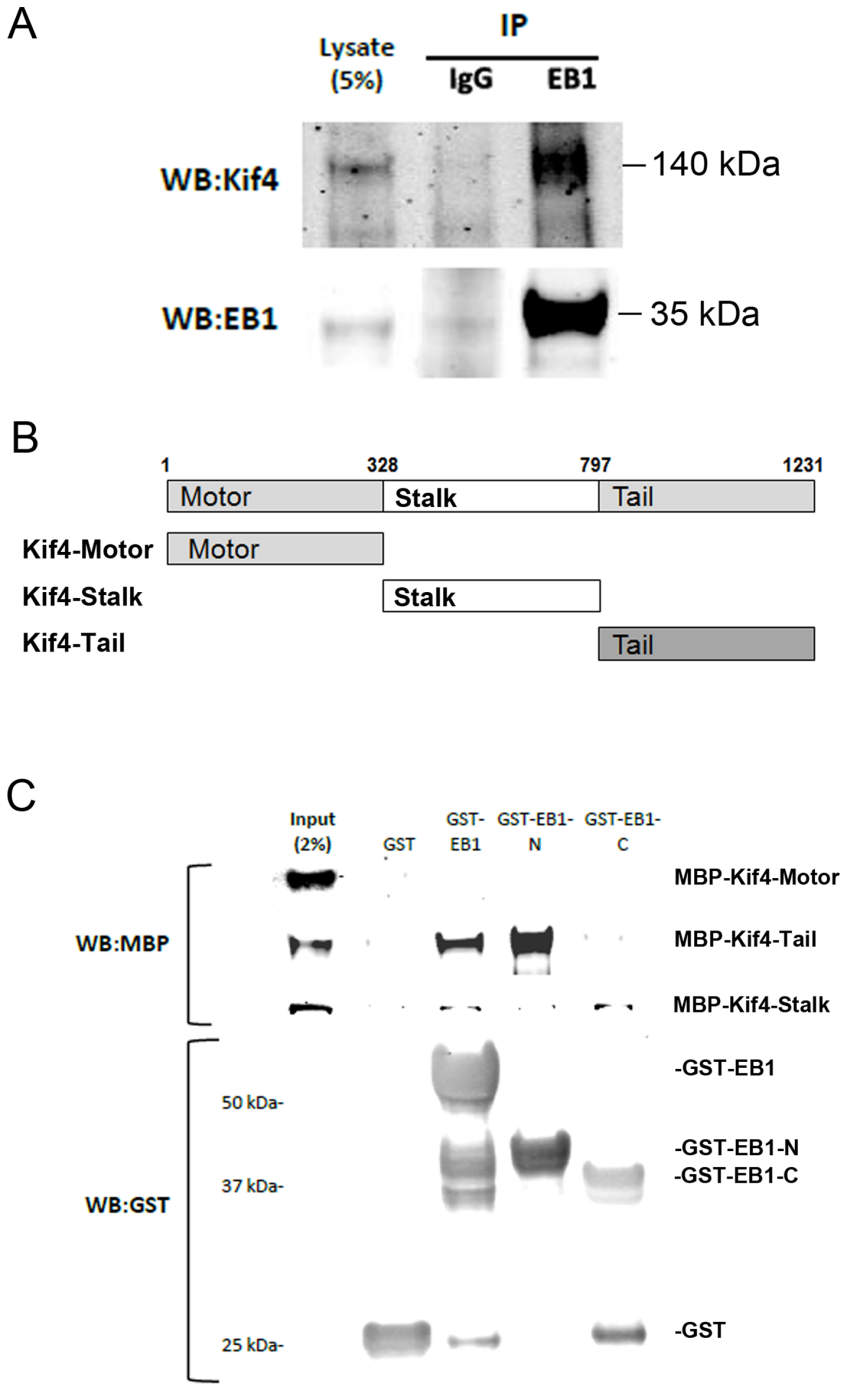
Kif4 interacts directly with EB1. A) Kif4 coimmunoprecipitates with EB1 from NIH3T3 fibroblast lysates. EB1 or control IgG immunoprecipitates were western blotted for EB1 and Kif4. B) Diagram of Kif4 protein fragments used for binding studies. C) Pull-down of recombinant proteins. Equal amounts of GST or the indicated GST-EB1 proteins on glutathione-Sepharose were used to pull down MBP-tagged Kif4 proteins. Bound proteins were analyzed by western blotting as indicated.

Given the mutual dependence of Kif4 and EB1 in inducing Glu MTs, we tested whether the proteins might interact. Immunoprecipitation of endogenous EB1 revealed that endogenous Kif4 associated with EB1 in NIH3T3 fibroblast lysates (Figure 6A). Kif4 has predicted N-terminal motor, central stalk with coiled coils domains and C-terminal tail domains (Figure 6B) [64]. Using purified recombinant proteins, we found that EB1 interacted directly with the tail of Kif4, but not the motor domain; there was also a weak interaction of EB1 with the stalk domain of Kif4 (Figure 6C). Using fragments of EB1, we found that Kif4 tail bound to the N-terminal domain of EB1, but not the C-terminal domain (Figure 6C). These results show that Kif4 associates directly with one of the previously established factors in the pathway for selective stabilization of MTs.

### Kif4 is necessary for efficient migration of cells

**Figure 7:**
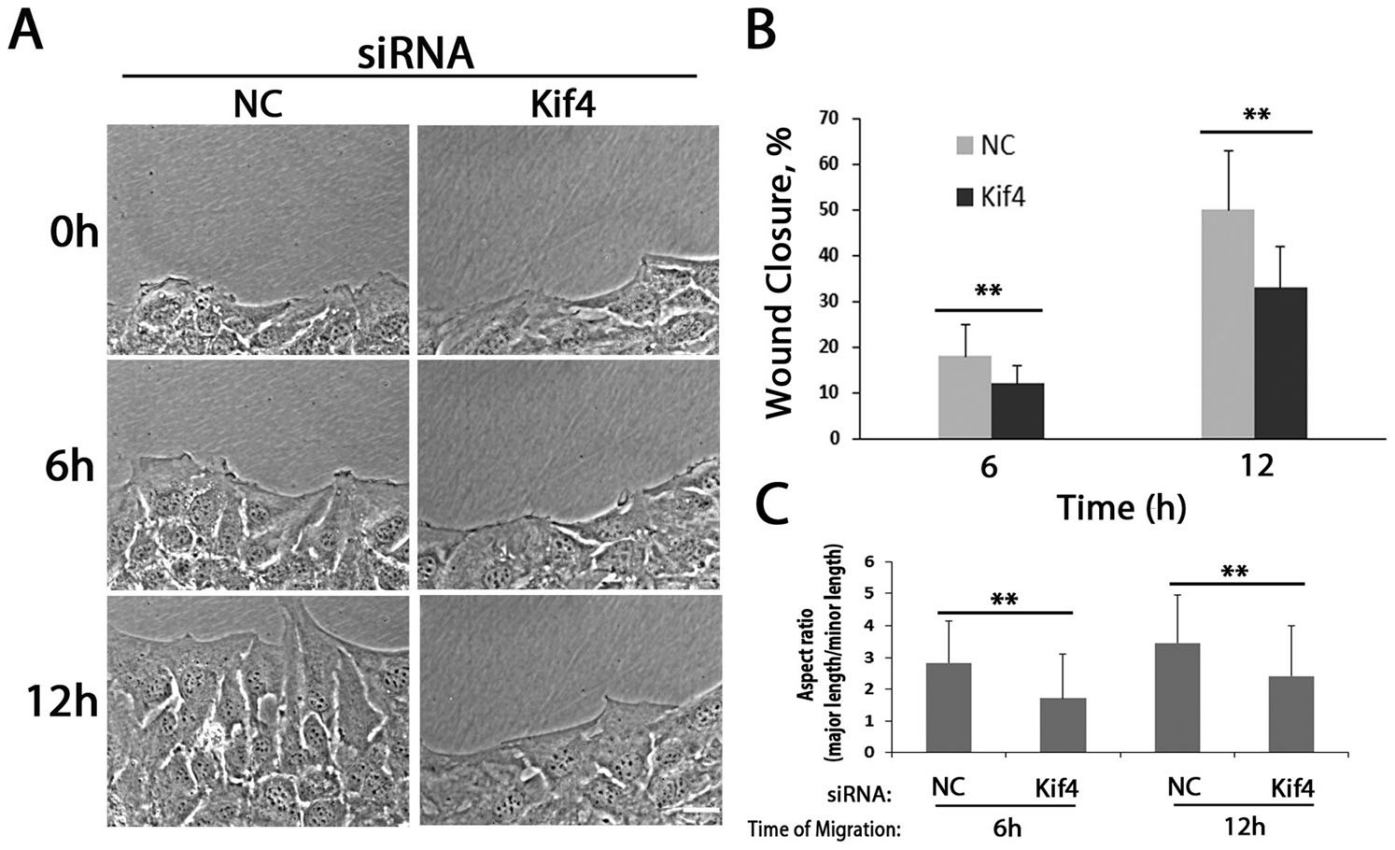
Kif4 knockdown inhibits cell migration into wounded monolayers. A) Panels from phase movies of wounded monolayers of NIH3T3 fibroblast treated with noncoding (NC) or Kif4 siRNAs. Bar, 15 µm. B) Quantification of the migration of NIH3T3 fibroblast monolayers after treating with noncoding (NC) or Kif4 siRNAs. C) Quantification of the cellular aspect ratio of wound edge NIH3T3 fibroblast treated with noncoding (NC) or Kif4 siRNAs and allowed to migrate for indicated times. Histograms in B, C are based on data from 3 experiments; error bars are SD.

Selectively stabilized MTs have been implicated in cell migration [17], [42], [65]. To test whether Kif4 contributed to cell migration, we knocked it down and measured rates of migration of NIH3T3 fibroblasts into in vitro wounds. Cells depleted of Kif4 still formed a normal confluent monolayer, but migration into the wound was reduced about 40% (Figure 7A, B). Analysis of the cell aspect ratio, a measure of overall cell polarization, revealed that Kif4 depleted cells had a significantly reduced aspect ratio compared to controls (Figure 7C). These results are consistent with earlier studies suggesting that stable MTs in the lamella contribute to cell migration by enhancing cell polarization and strengthen the notion that Kif4 has non-mitotic functions.

## Discussion

Previous studies revealed that long-lived Glu MTs in TC-7 cells and NIH3T3 fibroblasts exhibit the unusual property of not growing or shrinking for long intervals [33], [34], [42], [51]. This nondynamic behavior of Glu MTs contrasts with the bulk of the MTs, which undergo dynamic instability and exhibit much more rapid turnover. Glu MTs in detergent extracted TC-7 cell models behave as if they are capped at their plus ends by an ATP-sensitive activity that has characteristics of kinesin motors [51]. In this study, we identified Kif4 as a kinesin that is necessary and sufficient for the induction of Glu MTs and nocodazole resistant MTs in NIH3T3 fibroblasts. Our results indicate that Kif4 functions in the Rho-mDia-EB1 MT stabilization pathway because Kif4 depletion prevented the formation of Glu MTs in response to extracellular (LPA) and intracellular factors (DAD, LiCl and EB1) that activate this pathway, overexpression of Kif4 motor domain was sufficient to induce Glu MTs and Kif4 interacted with EB1, a previously identified factor in the pathway. Kif4 also localized to Glu MT ends where other factors in this pathway have been localized [42]. Our data suggest a model in which Kif4 contributes to the nondynamic behavior and stability of Glu MTs potentially by accumulating on Glu MT ends. Because only a subset of Glu MT ends had detectable Kif4 localization, we cannot rule out a model in which Kif4 may act more transiently, perhaps by transporting another factor in the pathway.

How does Kif4 stabilize MTs? Studies have identified two activities for Kif4. A motor function for Kif4 in the delivery of L1 cell adhesion molecule was described in studies of rat neurons [66], [67]. Kif4 also appears to be required for transporting Gag protein from murine leukemia virus and HIV [68], [69], the ribosomal protein P0 [66], and the mitotic protein PRC1 [70]. A MT stabilizing activity of the *Xenopus* Kif4, XKLP1 was identified in in vitro studies [56]. In this study, the motor domain of XKLP1 alone was shown to prevent the assembly and disassembly of dynamic MTs in vitro. Three pieces of data from our study are consistent with Kif4 generating nondynamic stabilized MTs through its predicted stabilizing activity: 1) induction of stabilized MTs by Kif4 motor domain, 2) localization of Kif4 on Glu MT ends, and 3) the ability of Kif4 to function downstream of other factors in the Rho-mDia MT stabilization pathway. Such a role would also be consistent with Kif4′s reported role in cytokinesis where it contributes to the stability and nondynamic nature of midzone MTs [57], [61], [70]. Additional studies will be needed to test whether mammalian Kif4 exhibits the direct MT stabilization activity of XKLP1 and/or whether Kif4 transport activity is necessary for MT stabilization.

Given the potent stabilizing activity of the Kif4 motor domain shown in the study of XKLP1, an interesting question arises in the context of selective stabilization of interphase MTs: how is the stabilizing activity of the motor regulated so that it selectively stabilizes only a subset of MTs in vivo? One possibility is that other factors in the Rho-mDia-EB1 pathway restrict its activity to specific locations. Rho is activated near the leading edge of migrating fibroblasts [71], but as yet there is no evidence that Rho or mDia interact with Kif4. Another possibility is that EB1 interaction with Kif4 may regulate its stabilizing activity. The yeast EB1, Mal3, interacts with the kinesin Tea2, and this interaction activates its motor activity [72]. The mammalian kinesin-2, Kif17, stabilizes MTs in epithelial cells in part by binding to EB1 [54]. A number of destabilizing kinesin-13s also interact with EBs and this interaction targets their activity to the MT plus end [73]. Perhaps, the stabilizing activity of Kif4 needs to be targeted to or retained on MT plus ends and this is accomplished by EB1. We note that in addition to this possible role for EB1, it is likely that EB1 plays a Kif4-independent role in MT stabilization, since EB1 interacts with a number of other components implicated in MT stabilization including mDia [42] and CLASPs [74].

Kif4 may also be regulated by phosphorylation, as has been shown for other kinesins [75]. PKCε is activated and GSK3β is inactivated downstream of mDia activation in fibroblasts and both contribute to formation of stabilized Glu MTs [63]. The downstream substrates of these kinases in the Rho-mDia stabilization pathway have not been identified. Kif4 has 12 known phosphorylation sites as shown by mass spectroscopy and two of these are predicted to be sites for GSK3β (S1017 and S1186; http://scansite.mit.edu/) [76]. Kif4 was recently shown to be activated by Aurora B phosphorylation in mitotic cells [77]. It would be interesting to test whether it is phosphorylated by one of these kinases during generation of stable MTs in LPA stimulated cells.

Kif4 has a well characterized role in cell division but there is growing evidence that Kif4 has roles in non-dividing cells (see references above). Our results show that even in serum-starved cells in G_0_ there is a small pool of cytoplasmic Kif4 and that cytoplasmic Kif4 increases with either LPA or serum stimulation. Consistent with a role in regulating interphase MT stability, we find that the axial polarization and migration of serum-stimulated cells was inhibited by Kif4 knockdown. Kif4 has a predicted nuclear localization sequence, yet we observed that nuclear accumulation of Kif4 was delayed for 12–24 h after serum-stimulation, suggesting that its nuclear localization is regulated in a cell cycle dependent fashion.

Members of the kinesin superfamily have been recognized for some time to participate in the regulation of MT dynamics in addition to their well-established role in acting as molecular transporters. Indeed, a subset of the kinesins, those in the kinesin-13 subfamily of which MCAK/Kif2 has been most intensively studied, are well-established MT depolymerases that recognize and promote the curved protofilament structure of depolymerizing MTs [78], [79]. The kinesin-8 family has also been implicated in regulating MT dynamics [80]. There are fewer kinesins that have been implicated in stabilizing MTs to generate long-lived and post-translationally modified MTs. Indeed, other than Kif4/XKLP1 the only other kinesin that has been reported to enhance MT longevity is Kif-17 [54]. In our study, we found that Kif17 was not expressed in NIH3T3 fibroblasts and expression of its motor domain did not induce MT stability in starved fibroblasts. Since the same construct induced MT stability in epithelial cells [54], these results suggest that different kinesins may be used to regulate MT stability in different cell types. It will be interesting to explore other kinesin subfamilies to determine whether there are other kinesins with the ability to generate long-lived, stable MTs.

## Materials and Methods

### Cell culture and chemicals

NIH3T3 cells (ATCC) were used throughout unless otherwise noted and were cultured in 10% calf serum in DMEM (Gibco BRL) as previously described [30], [34]. TC-7 cells (ATCC) were cultured as described previously [51]. MDCK cell lysate and Kif17 antibody (Sigma) were kind gifts from G. Kreitzer (Weill Cornell Medical College, NY). All chemicals were from Sigma-Aldrich unless otherwise noted.

### Cell starvation and LPA and serum treatments

NIH3T3 cells were passaged onto glass coverslips and after growing to confluency, were starved for two days in serum-free DMEM plus 10+ mM Hepes, pH 7.4 [30], [34], [52]. After wounding with a jeweler's screwdriver, MT stabilization was induced by adding 5 μM LPA for 2 hr. To examine Kif4 localization, starved NIH3T3 cells were stimulated for various times with either 5 μM LPA or 10% calf serum.

### Microinjection

Serum-starved NIH3T3 fibroblasts at the edge of wounded monolayers were pressure-microinjected with a micromanipulator (Narshige International). DNA (50 μg/ml) was injected into nuclei and recombinant protein (90 μM) was injected into the cytoplasm. After microinjection, the injected plasmid was allowed to express for 2 hr before fixation or further treatment with LPA.

### cDNA Constructs

Human GFP-Kif4 motor (residues 1–356), GFP-Kif3A motor (residues 1–354) and GFP-Kif17 motor (residues 1–335) were kind gifts of G. Kreitzer (Weil Cornell Medical College, NY). Mouse Kif4 full length was obtained from Open Biosystems and cloned into the Clontech GFP-C1 vector to prepare mouse GFP-Kif4. Human Kif4 fragments were subcloned into a maltose binding protein (MBP) vector pMAL-c2E (New England Biolabs) from the GFP-C1 vector after digesting with EcoRI and SalI and were verified by sequencing.

### Binding of purified proteins

Recombinant GST-EB1, GST-EB1-N and GST-EB1-C proteins were previously described [42]. MBP-tagged Kif4 proteins were expressed in Rosetta-2 bacteria (EMD Biosciences) and purified according to manufacturer's recommendations except using a different buffer (20 mM Hepes buffer, 150 mM NaCl, pH 7.5). Binding studies were performed by incubating 0.3 μM MBP-tagged Kif4 proteins in RIPA buffer (20 mM Tris pH 7.5, 150 mM NaCl, 1% Nonidet-P40, 1% sodium deoxycholate, 0.1% SDS) with 0.3 μM GST-tagged EB1 proteins on glutathione-agarose overnight at 4°C. After washing, bound proteins were eluted with SDS sample buffer and western blotted.

### Immunoprecipitation

Immunoprecipitation was performed overnight at 4°C using pre-cleared NIH3T3 fibroblast lysates in 10% RIPA buffer plus protease inhibitor cocktail (Sigma Aldrich) and MG132 (A.G. Scientific) and 1 μg rabbit anti-EB1 antibody (Santa Cruz Biotechnology) or non-immune rabbit IgG as a control. MG132 was critical to prevent degradation of Kif4 during the incubation. Immunoprecipitates were recovered with Protein A/G beads (1∶1 mix), washed, and the bound protein eluted with SDS sample buffer and analyzed by western blotting.

### siRNA transfection

Cells were transfected with siRNA (5 μM) using Lipofectamine RNAiMax (Invitrogen) according to manufacturer's instructions and plating cells directly into the transfection media. After overnight transfection, cells were serum-starved for 2 days. siRNAs were designed using the BIOPREDsi website (www.biopredsi.org/) and were obtained from Shanghai GenePharma. siRNA sequences were as follows: GAPDH, 5′-AAAGUUGUCAUGGAUGACCTT-3′; Kif4#1, 5′-GGAACUGGAGGGUCAAAUATT-3′; Kif4#2, 5′-GCAGAUUGAAAGCCUAGAGTT-3′; KIF3a, 5′-CAGGAAAUAACAUGAGGAATT-3′; EB1, 5′-CUGCCAGACAAGGUCAAGAAA-3′ *Fixation*. For routine immunofluorescence and for staining Kif4 in NIH3T3 fibroblasts, cells on glass coverslips were fixed in methanol for 5 min at −20°C. To detect Kif4 on the ends of MTs in TC-7 cells, cells were cryofixed in isopentane cooled in a dry ice-liquid nitrogen bath with constant stirring until it started to become gel-like, which corresponds to approximately −200°C [81]. After one min in isopentane, the cells were freeze substituted in a 6∶4 acetone:methanol on dry ice and then transferred to −80°C for 2 days. The cryofixed cells were gradually warmed by placing them in an insulated container at −20°C container for 4 hr and then transferred to TBS buffer (20 mM Tris pH 7.4, 150 mM NaCl) and stained with antibodies.

### Immunofluorescence staining

Rabbit anti-Glu-tubulin antibody (dilution 1∶400) was described previously [82]. Rat anti-Tyr tubulin (dilution 1∶10 of culture supernatant) was from European Collection of Animal Cell Cultures. Kif4 monoclonal antibody (dilution 1∶50) was described previously [66] and was a gift from A. Caceres or was from Sigma-Aldrich. Mouse anti-GFP antibody (dilution 1∶200) was from Sigma-Aldrich. Cyclin B antibody (dilution 1∶100) was from Santa Cruz. Secondary antibodies, absorbed to minimize interspecies cross reactivity, were from Jackson ImmunoResearch and were used as described previously [34]. Because most serum-starved NIH3T3 fibroblasts have a small number of Glu MTs before LPA stimulation, we scored cells as positive for Glu MTs if they had more than five brightly and continuously labeled Glu MTs that extended toward the cell periphery [30], [52]. The stability of MTs in cells expressing kinesin motors was tested by treating cells with 10 μM nocodazole for 30 min as described previously [30], [83].

### Western blotting

Samples were run on 4–15% polyacrylamide SDS gels and transferred to nitrocellulose. Blots were incubated with the following antibodies: anti-Kif4 mAb (1∶500), anti-beta-catenin (1;1000), anti-actin (Ab-5; 1∶10,000), anti-Kif3A (1∶100; BD Transduction Laboratories), anti-EB1 (1∶10,000), anti-Kif17 (1∶1000) or anti-GAPDH (1;6000). After incubation with fluorescent IR680- or IR800-conjugated secondary antibodies (1∶5,000, Rockland Immunochemicals), reactivity was documented with an Odyssey scanner (Li-Cor Biosciences).

### Analysis of Kif4 localization at Glu MT ends

NIH3T3 fibroblasts or TC-7 cells immunofluorescently stained for Glu tubulin, Tyr tubulin and Kif4 were mounted in TBS and imaged by TIRF microscopy on a Nikon TE2000 microscope with a 60X, 1.45 objective and an Orca II ER CCD (Hamamatsu) controlled by MetaMorph software. To unambiguously identify Glu MT ends, we overlaid Glu and Tyr MT images and considered only ends of Glu MTs in which the Glu tubulin staining abruptly stopped and was not “continued” with Tyr tubulin staining. Circles (10 pixels in diameter) were then drawn on the Glu MT ends and the overlaid on the corresponding image of Kif4 staining. Kif4 puncta inside circles that also contacted Glu MT ends were counted positive; those lacking Kif4 were counted negative. To control for random overlap of Kif4 puncta with Glu MTs, the circles were shifted 10 pixels in the x- and y-axes and the number of Kif4 puncta appearing in the circles was determined.

### Analysis of cell migration

Wounded monolayers of NIH3T3 fibroblasts treated with noncoding or Kif4 siRNAs were imaged with a 20X ELWD Plan Fluor objective (NA 0.45) at multiple positions every 10 min for 12 h on a Nikon TE300 microscope with a temperature controller (37°C) and motorized xyz stage. The extent of cell migration was measured as percentage of wound closure. The polarization of the cells was measured by determining their aspect ratio (major axis/minor axis) using Image J software.

### Statistical anaylsis

Statistical significance was assessed by Chi square analysis for non-parametric data and paired t-test for parametric data (Fig. 7C). P values are indicated in the figures as: *, p<0.05; **, p<0.01; ***, p<0.001.

## Supporting Information

File S1 **Supporting figures.**
(PDF)
