# Supplementary material for: Kif4 Interacts with EB1 and Stabilizes Microtubules Downstream of Rho-mDia in Migrating Fibroblasts
Source: PLoS One. 2014 Mar 21;9(3):e91568. doi: 10.1371/journal.pone.0091568 (PMC3962350; doi:10.1371/journal.pone.0091568)
Supplement: File S1 — Supporting figures. (PDF) [file pone.0091568.s001.pdf]

Supplemental Material, Morris et al.

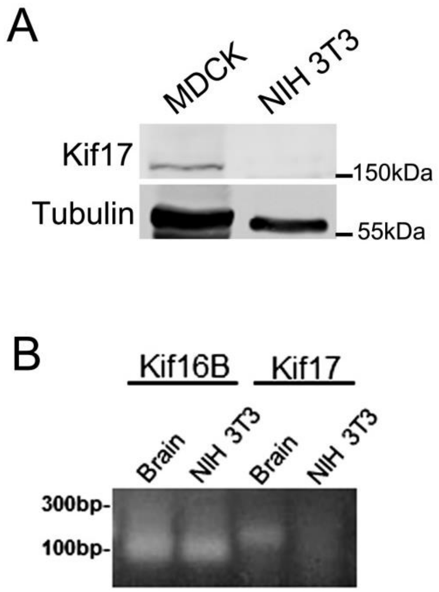

Figure S1. Analysis of Kif17 expression in NIH3T3 fibroblasts. A. Western blot analysis of Kif17 in MDCK cell and NIH3T3 fibroblast lysates. Tubulin is shown as a loading control. B. RT-PCR analysis of Kif17 expression in mouse brain and NIH3T3 fibroblasts. Kif16B is shown as a positive control.

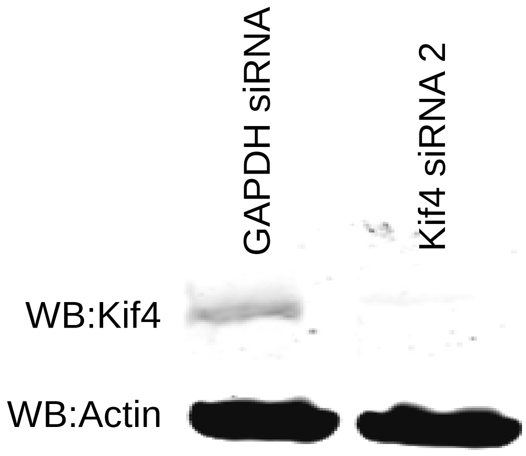

Figure S2. Western blot of Kif4 knockdown using a second siRNA. NIH3T3 fibroblasts treated with noncoding GAPDH or Kif4 siRNA #2 were analyzed by western blot. Actin was used as a loading control.

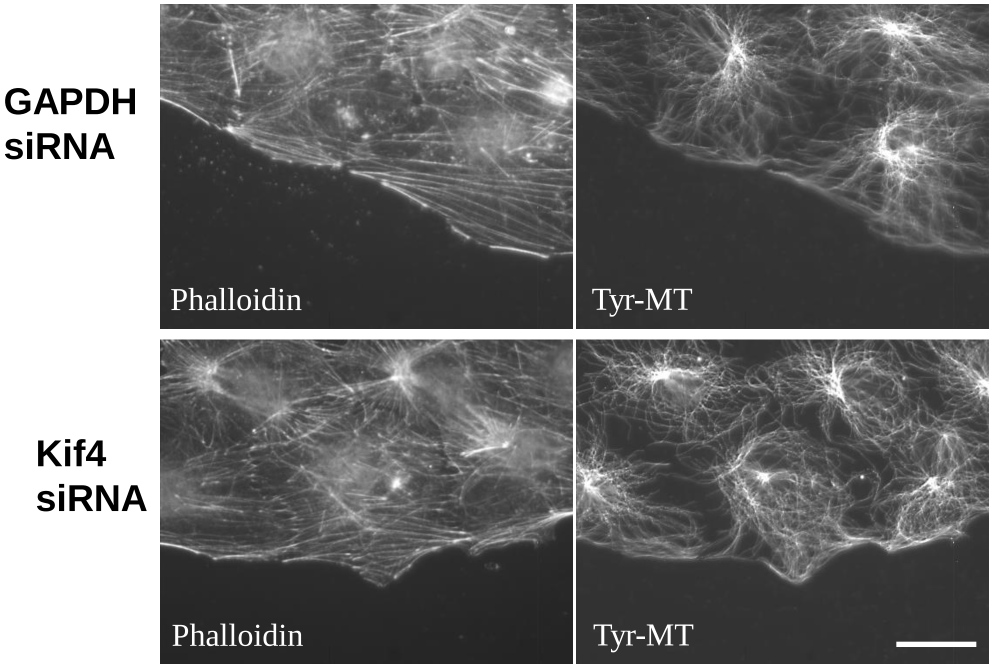

Figure S3. LPA stimulates normal actin fiber formation in Kif4 siRNA-treated NIH3T3 fibroblasts. Fluorescence images of F-actin stained with rhodamine phalloidin in LPA stimulated NIH3T3 fibroblasts that had previously been treated with control (GAPDH) or Kif4 siRNAs. Bar, 10 µm.

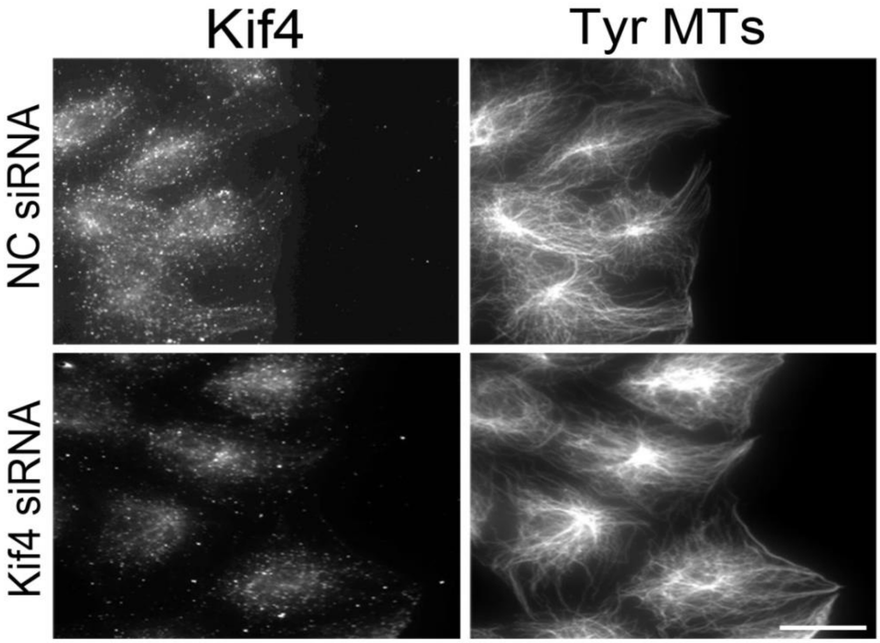

Figure S4. Immunofluorescence images of Kif4 and Tyr MTs in noncoding (NC) and Kif4 siRNA-treated NIH3T3 fibroblasts after LPA stimulation for 60 min. Note the reduction in cytoplasmic Kif4 immunostaining in cells treated with Kif4 siRNA. Bar, 20 µm.

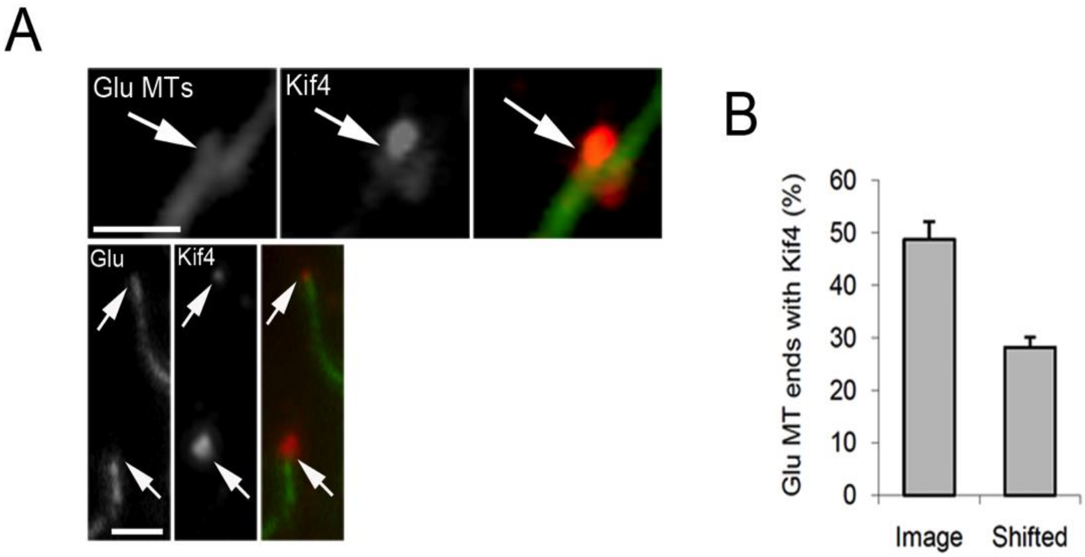

Figure S5. Kif4 localization on Glu MT ends in TC-7 cells. A) Immunofluorescence images of Kif4 and Glu MTs in TC-7 cells showing typical examples of localization of Kif4 on the ends of Glu MTs (arrows). B) Quantification of the percentage of Glu MTs ends that exhibit Kif4 puncta before and after shifting the Glu MT and Kif4 images relative to each other. n>20 ends, error bars are SEM from at least 3 experiments. Bar, 0.5 μm.

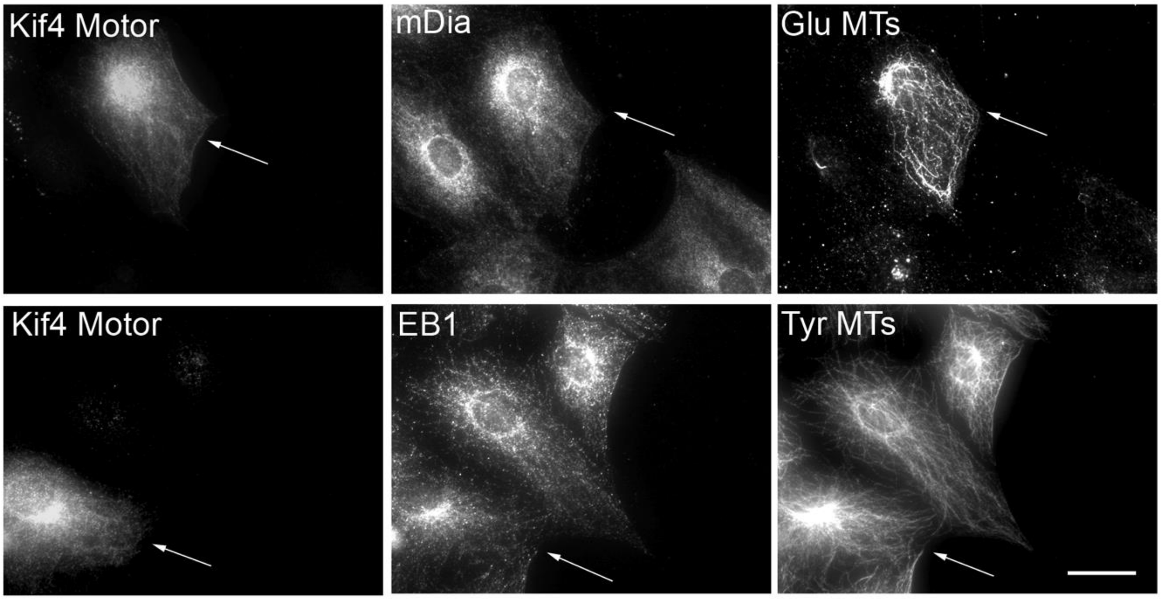

Figure S6. GFP-Kif4 motor expression does not change the distribution of mDia1 or EB1. Immunofluorescence of GFP, mDia1 and Glu MTs (top) and EB1 and Tyr MTs (bottom) in starved NIH3T3 fibroblasts expressing GFP-Kif4 motor. Arrows indicate GFP-Kif4 motor expressing cells. Bar, 10 μm.

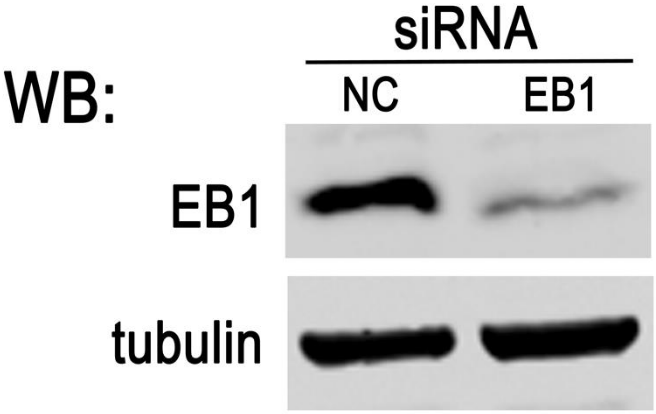

Figure S7. Western blot of EB1 knockdown using EB1 siRNA. NIH3T3 fibroblasts treated with noncoding (NC) or EB1 siRNA were analyzed by western blot. Tubulin was used as a loading control.
